# Development and Characterization of a Single Nucleotide Polymorphism Genotyping Panel for Duck Populations

**DOI:** 10.3390/ani16131995

**Published:** 2026-06-28

**Authors:** Yeongkuk Kim, Jaegwon Kim, Eunjin Cho, Seona Kwon, Minjun Kim, Hyojun Choo, Jun Heon Lee, Dongwon Seo, Jung-Woo Choi, Won-Hyong Chung

**Affiliations:** 1 Quantomic Research & Solution Co., Daejeon 34134, Republic of Korea; lgs10kr@naver.com; 2 Department of Animal Science, College of Animal Life Sciences, Kangwon National University, Chuncheon 24341, Republic of Korea; wornjsus@kangwon.ac.kr (J.K.); kwonseona23@gmail.com (S.K.); 3 Department of Bio-AI Convergence, Chungnam National University, Daejeon 34134, Republic of Korea; flunecmer@naver.com (E.C.); junheon@cnu.ac.kr (J.H.L.); 4 Poultry Research Institute, National Institute of Animal Science, Rural Development Administration, Pyeongchang 25342, Republic of Korea; mjkim6023@naver.com (M.K.); hyojy@korea.kr (H.C.); 5 Department of Animal Science and Biotechnology, Chungnam National University, Daejeon 34134, Republic of Korea; 6 TNT Research Co., Anyang 14059, Republic of Korea; dwseo@tntresearch.co.kr

**Keywords:** ducks, SNP, WGS, DNA genotyping panel

## Abstract

Single nucleotide polymorphism (SNP) genotyping panels for domesticated ducks are currently lacking, despite their importance for genetic diversity studies and genomic selection. We analyzed whole-genome sequencing data from 74 ducks. To obtain a 40K SNP marker set, we performed SNP calling and applied minor allele frequency (MAF) and linkage disequilibrium (LD) criteria. SNPs from 33 genes associated with Quantitative Trait Loci (QTL) were manually selected and added to the panel. The performance of the resulting SNP panel was validated using genotyping data from 28 additional duck samples. We developed a 40K SNP genotyping panel for ducks. Applying criteria of call rate, MAF, and LD, 37,386 SNP markers were selected. After excluding SNPs near repeats and indels, and incorporating 591 SNPs from 33 duck QTL-associated genes, the final set consisted of 35,613 SNP markers. When evaluating the performance of the SNP panel, genotypes were successfully called for 35,209 out of 35,613 SNPs, achieving a call rate of 98.9%. After applying QC filters, 33,870 SNPs were confirmed to be usable. Our SNP panel design process successfully generated a dataset at a 40K scale without compromising the genetic distances of the whole genome. By minimizing variation at the SNP probe alignment sites, we achieved a high call rate. The panel demonstrated reasonable robustness and suitability for further genomic studies, making it a valuable tool for genetic improvement and trait selection in duck populations.

## Simple Summary

Domesticated ducks are economically important poultry species raised for meat, eggs, and down worldwide, yet no commercial single nucleotide polymorphism (SNP) genotyping panel has been available for genomic studies in ducks. This study developed and validated a 40K SNP genotyping panel for domestic duck populations, including the Korean Native Duck. Using whole-genome sequencing data from 74 ducks spanning four breeds—the Korean Native Duck, Pekin Duck, Shanma Duck, and Shaoxing Duck—millions of SNPs were identified and filtered by quality, minor allele frequency, and linkage disequilibrium criteria. SNPs from 33 genes associated with economically important traits such as growth, pigmentation, and reproduction were additionally incorporated. The final panel of 35,613 SNPs was validated using 28 independent duck samples and achieved a genotyping call rate of 98.9%, with 33,870 SNPs confirmed usable after quality control. This panel provides a valuable resource for genetic diversity studies, genomic selection, and trait mapping in duck populations, and will support improvement of both indigenous and commercial duck breeds globally.

## 1. Introduction

The domestic duck (*Anas platyrhynchos domestica*) has been raised mostly for meat, eggs, and oil for human consumption and as a source of feathers and down [1]. Over 130 breeds of ducks are raised globally [2], as ducks are hardy, environmentally adaptable, and highly resistant to fatal infectious diseases, such as highly pathogenic avian influenza [3]. The origin of the domestic duck remains controversial. However, several recent studies have shown that many domestic duck breeds raised commercially in China, the country that consumes the most ducks in the world, originated from the Mallard duck (*Anas platyrhynchos*), with some introgression from spot-billed ducks (*Anas poecilorhyncha*) [4]. Domestic ducks have more varied plumage colors, and their flight is significantly limited compared to wild ducks, partly due to rearing environment practices aimed at greater meat or egg production, as well as the practice of cutting the wing tips. Overall, feral and currently domesticated ducks differ significantly in terms of their sizes and shapes, suggesting variations in their genomes.

The global production of duck meat is 5,516,351 tons, accounting for approximately 3.5% of the total meat production worldwide. Most of this production occurs in Asia, with China producing approximately 88% (as of 2022) [5]. Because duck meat consumption is increasing, especially in Asia, interest in duck meat production is growing in the United States and Europe as a potential export item to Asian markets [6].

In South Korea, two major biological duck types are raised: laying and broiler ducks. Egg-laying ducks are relatively rare, whereas broiler duck breeds account for 91.41% of the total South Korean duck production [7]. The Korean duck meat industry relies predominantly on imported duck breeds, mostly Pekin ducks (a meat-type breed), which are known for their fast growth rate and the high palatability of ducklings. Although South Korea has some indigenous duck breeds, these breeds have lower productivity than the Pekin type. To improve the performance of indigenous ducks, the National Institute of Animal Science (NIAS, Republic of Korea) collected ducks maintained in local farms and established pure lines of Korean Native Ducks (KNDs). Based on these pure lines, NIAS developed a practical crossbred strain, the “Woorimat” duck, which can be utilized mainly for meat production [8,9,10]. KNDs have been selected for several favorable traits at NIAS in South Korea since 1997 and are reported to have superior meat quality and a higher content of unsaturated fatty acids in breast meat compared with imported commercial ducks [11].

This selective breeding of Korean ducks is now establishing a reference population, thereby necessitating the introduction of genomic selection to increase the rapidity and efficiency of improvements in productivity [12]. In particular, the Korean duck industry requires improvement of productivity traits, including carcass weight and egg production, but it also needs the identification of useful traits with low heritability for future market differentiation. Accumulating large-scale genomic data for use in genetic improvement is expected to enable a multifaceted approach to setting future improvement goals [13,14,15]. Several previous studies have used genomic technology to improve the genetic merits of various livestock species. For example, some cattle or pig populations have shown efficient genetic improvement following the application of genomic selection using genotypic data derived from bead-chip arrays and whole-genome resequencing data [16].

Several genomic marker resources have also been reported in ducks. For instance, genotyping-by-sequencing (GBS) has been applied to Pekin ducks for genome-wide Single nucleotide polymorphism (SNP) discovery and genotyping [17]. Additionally, liquid-phase SNP arrays have been developed for specific commercial lines [18,19], along with medium-density SNP chips for commercial Pekin duck populations [15,20]. These advancements indicate that genomic marker platforms for ducks are being actively developed. However, SNP panels optimized for Korean Native Ducks and related domestic duck populations remain limited. Therefore, this study aimed to develop and validate a practical medium-density SNP panel for domestic duck populations, with particular emphasis on KND, by using WGS-based SNP discovery, genome-wide marker distribution, probe design evaluation, and incorporation of trait-associated gene markers.

The development of SNP chips for livestock species has greatly benefited from advancements in next-generation sequencing (NGS) technology, particularly whole-genome sequencing (WGS). For example, the BovineSNP50 chip containing about 54K markers was developed for cattle [21]. SNP chips have also been developed for pigs [22] and chickens [23], facilitating genomic selection and trait mapping in these species. WGS enables comprehensive variant discovery across the entire genome, providing a rich dataset for SNP chip design. Unlike traditional methods that rely on preselected genomic regions or markers, WGS provides an unbiased approach for identifying genetic variations, including SNPs, insertions, deletions, and structural variants. This comprehensive detection ensures that the resulting SNP chip includes variants that are truly representative of genetic diversity within and across populations.

In the present study, we used whole-genome resequencing data from 74 duck individuals, including Korean native, Pekin, and Chinese ducks, to develop an SNP panel for domestic duck populations. As both KNDs and Pekin Ducks (PKDs) are meat-type breeds, we additionally included two egg-laying breeds, Shanma Ducks (SMDs) and Shaoxing Ducks (SXDs), to enable comparison between ducks bred for different purposes and to enhance genetic diversity in the analysis. A very large number of SNPs were detected across the genome, and downstream analyses, including biological annotation and bioinformatic parsing, retrieved a total of 35,613 SNPs distributed across the duck genome. Our validation of the potential SNP panel using additional duck individuals showed a high call rate. This panel provides a practical tool for genomic studies and breeding of ducks, offering a genomic resource that encompasses both indigenous and commercial duck breeds and is compatible with the Illumina BeadChip platform widely used across livestock species.

## 2. Materials and Methods

### 2.1. Sample Selection

In this study, we used whole-genome resequencing data from a total of 74 duck individuals (Table 1). Of these, 30 were male, 30 were female, and the sex of 14 individuals could not be determined (Supplementary Table S1). Prior to sample collection, the NIAS established a foundation population (F1 generation) by collecting genetic resources from KND lines, including those found in farms located in Jeollanam-do, to secure and represent the indigenous diversity of KND. A total of 44 duck individuals were derived from the KND foundation population established by NIAS. Of the samples, 54 ducks (44 KNDs and 10 PKDs) provided by NIAS were directly sampled using a blood sampling method. The blood samples and associated data used in this study were collected in accordance with the regulation and approved by the Experimental Animal Care and Research Ethics Committee of the National Institute of Animal Science, Republic of Korea (Approval No. NIAS2023-0617; date of approval: 22 May 2023). The KND samples are a domesticated breed originating from the hybridization of domestic ducks traditionally raised in Korea with wild mallards, resulting in adaptation to local environmental conditions at NIAS. Blood samples were collected from the brachial vein, and genomic DNA (gDNA) was extracted using the PrimerPrep Genomic DNA Extraction Kit (GeNetBio, Daejeon, Republic of Korea). The quality of the extracted gDNA was confirmed using a NanoDrop 2000c spectrophotometer (Thermo Fisher Scientific, Waltham, MA, USA). The whole-genome sequence data of twenty duck samples, including 10 SMDs and 10 SXDs, were obtained from a duck population study by Li et al. [24]. The accession information can be found in Supplementary Table S1.

### 2.2. Reference Genome Selection

To select a reference genome for duck SNP chip design, we evaluated four assemblies—ZJU 1.0, duckbase.refseq.v4, CAU_Pekin_2.0, and CAU_Laying_1.0—in terms of assembly level, genome size, availability of gene annotation, and sequencing technology (Supplementary Table S2) [25,26]. Two genomes, ZJU 1.0 and duckbase.refseq.v4, are at chromosome level, while the others are at scaffold level. The largest assembly is CAU_Laying_1.0 and the smallest is duckbase.refseq.v4. Only ZJU 1.0 provides gene annotation via NCBI Genome, whereas duckbase.refseq.v4 was generated using exclusively short-read sequencing. Based on these criteria, we selected ZJU 1.0 as the reference genome for this study.

### 2.3. Sequencing, Read Alignment, and Variant Calling

The sequencing process for KND and PKD was as follows. A paired-end gDNA library was prepared following the NGS library preparation protocol (TruSeq Nano DNA library prep; Illumina, San Diego, CA, USA), and 151 bp paired-end reads were generated using the Illumina NovaSeq 6000 sequencing platform (Illumina, San Diego, CA, USA). Read alignment of the 74 duck samples was performed by aligning the reads to the reference genome assembly ZJU1.0 (accession no. GCF_015476345.1) [27]. We used Burrows-Wheeler Aligner for short-read alignment (BWA) version 0.7.17 [28] as the alignment tool. The aligned BAM files were sorted and indexed using SAMtools (version 1.19) [29]. Duplicate reads were marked with MarkDuplicates, and variants were called with HaplotypeCaller in GVCF mode to generate per-sample gVCF files. These files were merged using CombineGVCFs, followed by joint genotyping with GenotypeGVCFs, all implemented using the Genome Analysis Toolkit (GATK) version 4.4 [30].

We retained variants located in autosomal chromosomes and chromosome Z for further analysis. Quality control was applied to the resulting variant files, removing variants that met the following criteria. For SNPs, positions failing to satisfy any of the following conditions were filtered out: QD < 2, MQ < 40, FS > 60, SOR > 3, MQRankSum < −12.5, ReadPosRankSum < −8.0. For indels, positions meeting the following criteria were filtered out: QD < 2, FS > 200, SOR > 10, ReadPosRankSum < −20, InbreedingCoeff < −0.8. We also selected SNPs satisfying the following options: TYPE = ‘snp’ and N_ALT = 1, to identify bi-allelic SNPs, and F_MISSING < 0.2, corresponding to a call rate ≥0.8.

### 2.4. Retrieving SNP Set for the Panel

To reduce the number of markers for the SNP panel, we measured SNP density and the degree of association between SNPs. We calculated the minor allele frequency (MAF) and linkage disequilibrium (LD) using PLINK and an in-house program. For the filtered SNPs, we chose SNPs showing MAF > 0.2 and LD value less than 0.4 R^2^ across the entire population. We then selected SNPs evenly across the genome based on LD pruning and physical distance using a custom script developed in-house. We then performed additional filtration, removing SNPs located within 100 bp of repeats or indels. Repeat information was obtained from the genome information stored in NCBI Genome, computed using RepeatMasker [31] (version 4.0.8) with the following parameters: “-engine wublast -species ‘anas platyrhynchos’ -s -no_is -cutoff 255”. Subsequently, SNPs were filtered through Illumina Design Studio (Illumina, San Diego, CA, USA), retaining only those with a design score >0.6.

### 2.5. Functional Annotation of the Identified Variants

We used SnpEff (version 5.2) [32] for functional annotation of the genetic variants. The genomic features in GFF format and the genome sequences in FASTA format were obtained from the Ensembl genome database to build the SnpEff database for the ZJU 1.0 genome. The building process was done using the ‘build’ command in SnpEff. The functional annotation was performed using SnpEff with the ‘-canon’ option. We identified genomic coordinates of 33 genes reported in ducks to be associated with quantitative trait loci (QTL) or economically important traits. We defined the range of variants affecting genes as the gene’s location plus 5 kb upstream and downstream regions. We then manually selected and added SNPs located in trait-associated genes.

### 2.6. Validation of SNP Panel

The genome-wide distribution of the final SNP markers was visualized using the CMplot package in R. SNP density was calculated as the number of SNP markers within non-overlapping 300 kb genomic windows across autosomes and chromosome Z. The density plot was used to evaluate the genomic distribution of the selected SNPs and to identify genomic regions with relatively high or low marker density.

A total of 28 additional KND samples were selected to validate the genotype detection performance of the developed SNP panel. These samples were independently collected from a separate population and were randomly selected, ensuring no overlap with the initial WGS dataset. For DNA extraction, 2 μL of blood from each sample was quantified, followed by the addition of lysis buffer and incubation in a shaking incubator at 65 °C overnight to dissolve nucleic acids. The automated genomic DNA extraction process used the KingFisher system (Thermo Fisher Scientific, Waltham, MA, USA). The extracted DNA was quantified and subjected to quality control using the EPOCH system (BioTech Inst. Inc., Washington, DC, USA) and stored at −20 °C. The 28 validation samples were genotyped using the newly developed Korean Duck SNP panel. Genotyping analysis was conducted using the Illumina iScan system (Illumina, San Diego, CA, USA) following the Infinium HD Assay Ultra Protocol, with 200 ng of gDNA used per sample. Analysis results were reviewed using GenomeStudio software (version 2.0.5; Illumina, San Diego, CA, USA) to verify the genotype call plots. Genotype data were extracted in PED and MAP file formats using the PLINK plugin.

## 3. Results and Discussion

### 3.1. Whole-Genome Sequencing

We identified genome-wide SNPs using whole-genome resequencing data derived from 74 duck individuals from four duck populations: KND, PKD, SMD, and SXD (Table 1). We included 44 KNDs in our SNP panel to ensure compatibility with the typical indigenous duck breeds found in South Korea. We also sequenced 10 PKDs as the de facto main broiler duck breed currently dominating the South Korean duck meat market. Using the Illumina NovaSeq 6000 sequencing platform, we obtained high-depth sequencing data with an average coverage of 40× per individual (Table 1; Supplementary Table S1). To ensure accurate and reliable variant discovery, we generated 16 billion paired-end reads, each with a length of 151 bp, resulting in approximately 2.5 terabases of sequencing data.

We downloaded sequencing data for SMD and SXD from the NCBI Sequence Read Archive (SRA). To ensure data quality, we selected datasets with a minimum coverage depth of 20× and at least 10 samples per breed (Table 1; Supplementary Table S1). As a result, 3.5 billion paired-end reads (150 bp each), totaling 525 Gb, were added. Combined with our newly sequenced data, approximately 3 Tb of whole-genome sequencing data were used for the analysis.

For the SNP genotyping analysis, we chose the ZJU 1.0 duck genome sequence assembly as the reference due to its relatively higher completeness compared to the other duck genome assemblies [27]. We evaluated four duck genomes: two Pekin duck genomes (duckbase.refseq.v4 and CAU_Pekin_2.0) and one Shaoxing duck genome (CAU_Laying_1.0) [25,26] in addition to ZJU 1.0 (Supplementary Table S2). The ZJU 1.0 genome appears to have superior assembly quality and completeness compared to the others, making it the most suitable reference genome for our analysis. This choice is further supported by a recent duck pan-genome study, which also selected ZJU 1.0 as the reference genome and reported that it had the lowest error rate and the highest consensus quality among the four assemblies [33].

### 3.2. Variant Calling and Genotyping

The generated sequencing reads were mapped to the reference genome, covering 97.8% of the genome assembly with an average mapping rate of 98.4% (Table 1). For the directly sampled KND and PKD breeds, 98.4% genome coverage and 99.3% mapping rate on average were achieved, while SMD and SXD breeds achieved 96.3% coverage with 96% mapping rate. The sequence statistics were sufficient to enable further identification of reliable genomic variations across the genome. After the initial mapping process, potential duplicate reads were marked, and the variants were called following the recommended process of GATK [30]. This process included generating a gVCF file for each sample, combining these into a single dataset, and performing joint genotyping to produce the final variant calls. These steps ensured a comprehensive and standardized approach to variant discovery. For sex chromosome Z, variant calling and genotyping were performed exclusively on the thirty male ducks used in this study (Supplementary Table S1).

As a result of the genotyping process, we identified a total of 33,464,919 variants (step 1, Table 2). Of the total identified variants, 80.8% were biallelic SNPs and 10% were biallelic indels. The remaining 9.2% were classified as multiallelic or complex variants. This distribution is similar to those observed in other domesticated animal species, indicating that the four duck populations used in this study (KND, PKD, SMD, and SXD) are genetically distinct but not highly divergent.

### 3.3. Variant Filtration

In this study, only 31 autosomes (chr1–29, chr31, and chr33) and a sex chromosome (chromosome Z) were considered. Although ducks have a karyotype of 40 chromosomes, the ZJU 1.0 assembly includes only 31 autosomes plus two sex chromosomes, indicating that this reference still lacks several chromosomes and is therefore relatively incomplete. We excluded the W chromosome because it is female-specific and male samples cannot be genotyped. The unplaced contigs were also excluded from the SNP chip design because their correct genomic locations have not yet been determined. The end result was 30,365,553 variants, representing 90.8% of the full dataset (step 2, Table 2).

Variant quality filtration was then applied with different criteria for SNPs and indels. This process retained a total of 28,414,251 variants consisting of 23,525,876 bi-allelic SNPs (82.8%), 2,916,668 indels (10.3%), and 1,971,707 complex variants (6.9%) (step 3, Table 2).

The presence of complex variants complicates genotyping due to the increased difficulty of accurately distinguishing between SNPs and indels at a single location. Indels can introduce alignment challenges, particularly in repetitive or structurally complex regions, leading to lower accuracy in variant calling. The size variability introduced by indels further complicates probe design for genotyping arrays, as the probes may fail to capture the full sequence context. Due to these limitations, both complex variants and indels were excluded from the marker candidates in further marker panel design.

At the initial discovery-level filtering step, biallelic SNPs were selected and SNPs with call rates lower than 0.8 were removed. This filtering step was applied to retain a sufficient number of candidate SNPs across the four duck breeds while excluding SNPs with excessive missing genotypes. As a result of the filtration process, we obtained 23,049,128 candidate SNPs (step 4, Table 2). These SNPs were distributed at an average interval of one SNP every 48.6 bp in the reference genome. This provided comprehensive coverage of the entire genome and was more than sufficient for further identification of genetic variations for use in developing a panel of SNPs evenly populated across the duck genome—one of the prerequisites for successful design of an SNP Bead-Chip array panel for genomic prediction of various traits.

### 3.4. Selection of SNP Markers for Panel Design

The number of markers used in the SNP marker panel was reduced by conducting a selection process based on MAF and LD of each SNP marker within the population. We used several parameters for duck genotyping refinement and conducted simulation testing to determine the most suitable selection criteria for MAF and LD. We determined the most appropriate MAF threshold using SNPs with MAF values above thresholds set from 0.1 to 0.3 (in increments of 0.05) to create five test sets, and the changes in the population structure were examined for each case. We determined the appropriate LD threshold for intervals of potentially 40K SNPs using R^2^ values ranging from 0.1 to 0.9 (in increments of 0.1) as thresholds and excluding markers that exceeded these criteria to create various marker sets. Based on the examination, we chose the SNP selection criteria as MAF > 0.2 and LD values of R^2^ < 0.4. These selection criteria are similar to those applied in the development of SNP chips for livestock and poultry species, in which SNP markers with MAF > 0.2 were selected to exclude low-frequency variants and ensure the reliability of the marker set [34], and LD pruning based on R^2^ thresholds has been applied to reduce redundancy among tightly linked SNP markers while maintaining uniform genomic coverage [23,35]. We applied filtration based on LD value to ensure the selection of high-quality and representative markers for the SNP chip. SNPs with an LD value of R^2^ less than 0.4 were selected, as lower LD values indicate less redundancy and greater independence among the SNPs. This step was critical to avoid overrepresentation of tightly linked SNPs in the chip design.

**Figure 1:**
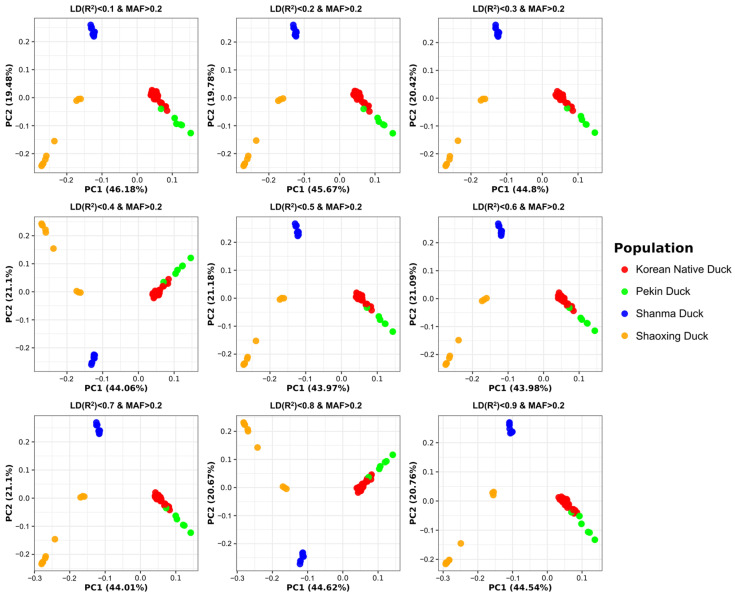
Principal component analysis (PCA) plot according to LD and MAF values. PCA plots represent the clustering of duck breeds based on LD and MAF values. The X-axis shows PC1, and the Y-axis shows PC2. Different colors indicate the four duck breeds, as shown in the figure legend.

**Figure 2:**
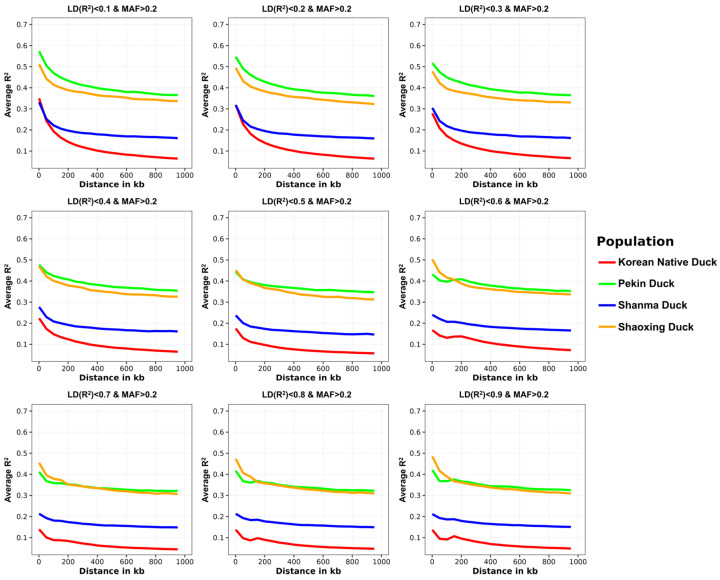
LD decay plot according to LD and MAF values. LD decay patterns for four duck breeds across varying LD thresholds (0.1 to 0.9). The X-axis represents genomic distance (kb), and the Y-axis shows r^2^ values. Different colored curves indicate the four duck breeds, as shown in the figure legend.

To evaluate whether our SNP-selection thresholds altered inferred structure, we performed PCA on candidate marker sets constructed under five MAF thresholds and nine LD pruning thresholds. Across all combinations, PCA consistently resolved the four populations (KND, PKD, SMD, and SXD) with stable group centroids and similar within-group dispersion, indicating that clustering was robust to both allele frequency filtering and LD pruning. In particular, varying the LD cutoff from 0.1 to 0.9 did not materially shift the relative positioning of the four groups on PC1–PC2, and increasing the MAF threshold from 0.1 to 0.3 did not show significant changes between the groups. Therefore, the PCA plots based on varying LD and MAF values revealed no significant changes in clustering or population structure under any criteria (Figure 1). The LD decay plot also demonstrated that an LD R^2^ value of 0.4 and an MAF threshold of 0.2 represented optimal filtering criteria, as they effectively explained the genetic structure and diversity. Genome-wide LD-decay curves computed within breeds and across all samples showed the expected monotonic decline in R^2^ with increasing inter-marker distance. When we compared panels generated under alternative pruning settings, more stringent thresholds removed a substantial number of loci yet did not improve population separation in PCA, whereas more lenient thresholds (e.g., R^2^ > 0.6) retained locally redundant markers. Choosing R^2^ = 0.4 therefore balanced the goals of reducing linkage redundancy and maintaining uniform genomic coverage, yielding a marker set that is informative for diversity and downstream applications (Figure 2). After filtering based on LD, we used an in-house program for further refinement of the selection. This program was designed to choose representative SNPs that were evenly spaced across the genome. The even spacing of SNPs is essential for achieving uniform coverage and ensuring that all genomic regions are adequately represented on the chip. The filtering process resulted in the selection of 37,386 SNPs as marker candidates for the 40K duck genotyping panel.

### 3.5. Removal of SNP Markers near Repeats and Indels

Despite recent achievements in sequencing and bioinformatics technologies, indels and repeat regions can pose challenges that affect the accuracy and reliability of genotyping results in DNA genotyping microarrays. Therefore, excluding markers that overlap with indels or repeat regions is preferable to ensure marker reproducibility for the SNP panel design. A previous study on the development of the 60K chicken SNP chip excluded SNPs in repetitive sequences and unassembled regions to enhance the chip’s accuracy and efficiency [23]. Similarly, a 200K SNP chip for endangered German cattle breeds used a similar approach to improve chip performance [36]. Moreover, when probe sequences overlap with indels or repeat regions, hybridization can become less specific and efficient. For these reasons, we excluded markers that were located within 100 bp of either indels or repeat regions. As a result, 1306 markers were filtered out, leaving a set of 36,080 candidate markers.

### 3.6. Addition of SNP Markers at QTL-Associated Genes

To enhance the functional relevance of the chip, we conducted a literature review to identify genes associated with economically important traits in ducks. We selected genes that have been reported in the literature to be associated with these traits. Functional annotation of the markers was performed using the SnpEff tool. As the reference genome ZJU1.0 lacked updated annotation information, we built a custom SnpEff database for the duck genome (ZJU 1.0 version) using a de novo approach. Using SnpEff, we annotated SNP markers in 33 genes associated with several important duck traits, such as pigmentation, growth, and reproduction (Supplementary Table S3).

The gene list includes several genes involved in growth and development (e.g., *IGF1*, *IGF2*, *IGF2BP1*, *GH1*, *VLDLR*, *PRLR*, and *SLC7A5*), which play key roles in regulating body size, muscle development, and metabolic activity [26,37,38,39,40]. Pigmentation-related genes (*MITF*, *TYR*, *MC1R*, *TYRP1, DCT*, *SOX10*, and *KIT*) are involved in the melanogenesis pathway, contributing to feather and skin color variation [14,41,42,43]. Reproductive trait genes (*ESR1*, *ESR2*, *RNF17*, *LHX9*, and *MC2R*), which are known to regulate hormonal activity and fertility, were included [37,44,45,46]. We also included genes associated with the nervous system and neurotransmission (*DRD2*, *GRIA1*, *DBH,* and *POU4F3*), reflecting their potential roles in behavior and stress response [38,45,46]. Additionally, regulatory and signaling-related genes such as *PARP4*, *ASAP1*, *IPMK*, *CENPJ*, and *STARD9* were included due to their involvement in transcriptional control and intracellular signaling pathways [40,44,47]. SAA2, which encodes serum amyloid A protein and is involved in the acute-phase immune response in ducks, was also included [48]. Finally, four putative genes (*LOC101798015*, *LOC101798291*, *LOC101805249*, and *LOC101805103*) were added to capture potential novel functional loci [40,44,46].

To more clearly capture these trait-associated genes, we manually curated markers within their genomic regions regardless of LD. As a result, 529 markers were successfully added, corresponding to approximately 18 markers per gene on average. A total of 36,609 chip-ready SNP markers were therefore obtained. A detailed list of SNPs located in the genic regions of the 33 genes is provided in Supplementary Table S4.

The 33 genes included in this study were selected based on previous reports of their association with economically important traits in ducks. Therefore, these genes should be considered literature-supported trait-associated genes, not functionally validated genes identified in the present study. Further validation using independent populations with trait records will be required to confirm the biological effects of the selected SNPs.

### 3.7. Microarray Assay Design for Ducks

As the final step, the SNPs were subsequently filtered through Illumina Design Studio, retaining only those with a design score >0.6 and yielding a final selection of 35,613 SNPs. Chromosome 1 had the highest number of variants, totaling 13,613, while Chromosome 33 had the fewest, with only 22 variants. One of the key explanations for the observed high LD values is the even distribution of distances between the selected SNPs. The selected SNPs were optimized for high LD values to improve the performance of the chip, and the distances between SNPs were evenly distributed to cover all chromosomes on the reference genome.

To further evaluate the quality of the final designed marker set, we performed an in silico marker-level call rate assessment using the discovery genotype dataset. All 35,613 final markers passed the gap-region and MAF criteria applied in the design process. Among these markers, 35,460 markers (99.57%) showed a call rate ≥0.85, 35,272 markers (99.04%) showed a call rate ≥0.90, and 34,882 markers (97.95%) showed a call rate ≥0.95. These results indicate that most markers in the final design panel had high genotype completeness in the discovery dataset, even under more stringent call rate thresholds.

Through this process, we obtained a final duck SNP marker set suitable for custom Illumina array design. Although the array format can technically accommodate both duck and chicken SNP markers, the present study focused only on the development and validation of the duck SNP markers.

### 3.8. Functional Annotation of the Duck SNP Panel

We annotated all 35,613 variants on the duck 40K SNP chip by biotype, impact, and genomic location (Supplementary Tables S5–S7). In the biotype distribution (Supplementary Table S5), protein-coding variants were most common (52.5%), followed by intergenic (39.0%) and lncRNA (8.4%), with all other biotypes totaling only 29 markers (0.1%). Impact predictions (Supplementary Table S6) showed 96.8% MODIFIER variants, 2.6% LOW, 0.7% MODERATE, and 0.01% HIGH. The genomic location of the SNPs (Supplementary Table S7) indicated that approximately 64.8% of variants lie within genic regions while 35.2% are intergenic. These annotations demonstrate that our SNP panel is enriched for functional genomic elements, while also providing broad coverage across the duck genome.

### 3.9. Validation of the Duck SNP Panel

**Figure 3:**
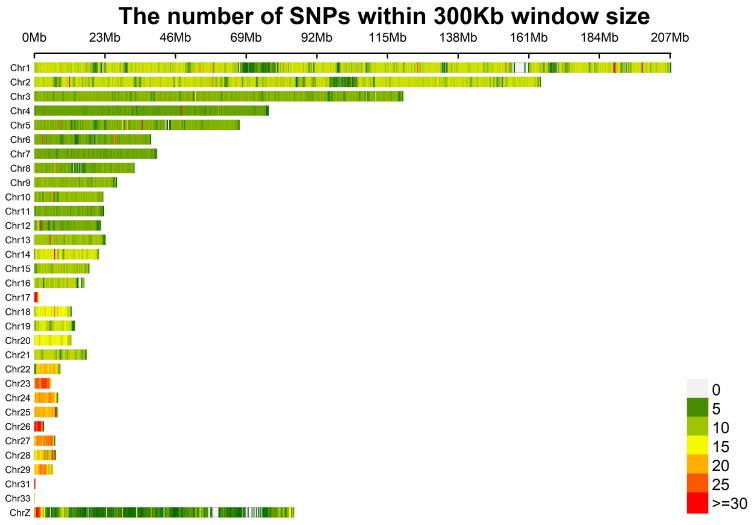
Genome-wide SNP density heatmap of the duck SNP panel. SNP density was calculated as the number of SNP markers within non-overlapping 300 kb genomic windows across autosomes and chromosome Z. The color scale indicates the number of SNPs per window, ranging from 0 to ≥30 SNPs.

We examined the genome-wide distribution of the final SNP markers using a SNP density heatmap (Figure 3). SNP density was calculated as the number of SNP markers within 300 kb genomic windows across autosomes and chromosome Z. The heatmap showed that the selected SNPs were broadly distributed across the duck genome, although local differences in marker density were observed among chromosomes and genomic regions.

Most genomic windows contained approximately 5–15 SNPs, indicating relatively even marker distribution across the genome. These low-density regions may reflect local filtering effects caused by LD pruning, MAF filtering, repeat/indel filtering, and Illumina probe design constraints. Short chromosomes tended to show more variable local density patterns because a small number of markers can strongly affect density estimates in short genomic regions. Some high-density regions, including those observed on chromosomes that were not short, were mainly related to QTL-associated gene regions. Because SNPs located in the 33 trait-associated genes were manually added to enhance the functional relevance of the panel, local increases in SNP density were expected in these genomic regions. Chromosome Z showed broad marker coverage, but several low-density windows were also observed.

When marker distribution was evaluated relative to the number of genes per chromosome, the SNP count per gene was relatively balanced across most chromosomes (Supplementary Figure S1). This suggests that the final panel retained broad genomic coverage while showing some regional density variation caused by both marker filtering and intentional inclusion of trait-associated loci. However, chromosome 31 showed a distinctly higher SNP density, which we suspect is a consequence of the incompleteness of the duck genome assembly.

To evaluate population structure, we performed pairwise FST analysis and constructed an SNP-based genetic distance tree. Pairwise FST values ranged from 0.079 to 0.307 among the four populations (Supplementary Table S8). The lowest FST was observed between KND and PKD (0.079), whereas the highest FST was observed between PKD and SXD (0.307). This indicates that KND and PKD are relatively close, while the egg-type populations are more differentiated from the meat-type populations. The SNP-based tree showed a similar pattern (Supplementary Figure S2). PKD individuals were positioned close to the KND cluster, suggesting a close genetic relationship between these two meat-type populations. In contrast, SMD and SXD were more clearly separated from the meat-type populations. Together with the PCA results in Figure 1, these analyses indicate that the selected SNP markers retain sufficient information to describe the major genetic relationships among the discovery populations.

We validated the genotyping performance of the SNP panel by collecting an additional 28 duck samples and extracting DNA for SNP genotyping. Of the 35,613 SNPs, genotypes were successfully identified for 35,209 SNPs, achieving a call rate of 98.9%. After applying QC filters in PLINK (geno > 0.1, MAF < 0.01, and removal of monomorphic SNPs), 33,870 SNPs were confirmed as usable. This indicates that only 1743 SNPs were filtered out after genotyping, demonstrating a highly positive outcome in terms of usability. During the initial design of Illumina’s chicken 60K SNP panel, 57,636 SNPs were selected and 54,293 (94.2%) proved usable after genotyping [23]. In contrast, our SNP chip designed for ducks achieved a superior conversion rate, with about 99% of markers yielding genotypes of high quality, demonstrating its enhanced performance.

Our SNP chip achieved nearly a 99% call rate, demonstrating the effectiveness of our SNP marker selection process. By comparison, Illumina’s chicken 60K panel originally included 57,636 markers but delivered a 94.2% call rate (54,293 usable SNPs), and the chicken 600K array produced just 68% reliably polymorphic markers [23,49]. Such a high call rate, compared to other avian Illumina panels, offers superior usability and efficiency for genetic studies in ducks. Compared to these results, the SNP panel developed in this study demonstrates a highly satisfactory level of usability. Although SNP discovery was performed using four duck populations, the technical validation of the panel was conducted only with independent KND samples. Therefore, the present results demonstrate the performance of the panel mainly in KND, and additional validation using independent non-KND samples will be required before broad application to other duck breeds.

The limited discovery sample size may be considered a potential source of ascertainment bias. Although the 74 ducks used for SNP discovery represented four breeds and included both meat-type and egg-laying ducks, this dataset may not fully capture rare variants, breed-specific variants, or private alleles in duck breeds not included in this study. Therefore, the present panel may be regarded as a foundational SNP resource for KND and related domestic duck populations. Further improvement and validation using additional breeds and geographically diverse populations may help increase its broader applicability.

## 4. Conclusions

This study developed and technically validated a 40K-level medium-density SNP genotyping panel for Korean Native Ducks and related domestic duck populations. The final panel contained 35,613 SNP markers and showed reliable genotyping performance in 28 independent KND samples, with 35,209 SNPs successfully called, corresponding to a call rate of 98.9%. After quality control, 33,870 SNPs were retained as usable markers. These results indicate that the marker selection strategy, including the removal of SNPs near indels and repetitive regions and evaluation of probe design suitability, was effective for producing a robust genotyping panel for duck genomic studies.

The SNP panel also provides a foundation for future genome-wide association studies, genomic prediction, and genomic selection in ducks by enabling the integration of genotype data with carcass, growth, egg-production, meat-quality, and other breeding records. This panel can be directly applied to KND breeding programs for routine genotyping of reference populations, assessment of genetic diversity and population relationships, pedigree and sample identity checking, and accumulation of genomic data for selection candidates. The inclusion of SNP markers from 33 trait-associated genes also increases its utility for investigating genomic regions related to economically important traits such as growth, pigmentation, and reproduction.

However, this study has several limitations, including technical validation only in independent KND samples, the limited discovery sample size, and the absence of phenotypic validation for the selected trait-associated candidate loci. Therefore, further validation using diverse duck breeds and independent populations with recorded phenotypes will be required to evaluate cross-breed applicability, reduce potential ascertainment bias, and confirm the biological effects of these markers. Future studies using larger trait-associated populations will further enhance the application of this SNP panel in duck breeding and genomic research.

## Figures and Tables

**Table 1:** Summary of duck breeds and sequencing data used for SNP chip design.

Breed (* Abbr.)	Population Size	Sequencing Reads (M)	Read Length (bp)	Sequencing Bases (Gb)	Sequencing Depth (X)	Mapping Rate (%)	Genome Coverage (%)
Korean Native Duck (KND)	44	13,718	151	2072	39.7	99.3	98.4
Pekin Duck (PKD)	10	3057	151	462	38.9	99.2	98.5
Shanma Duck (SMD)	10	1798	150	270	22.7	94.0	96.7
Shaoxing Duck (SXD)	10	1696	150	255	21.4	98.2	96.1
Total	74	20,267		3057	34.8	98.4	97.8

* Abbr.: three-letter abbreviation used for each duck breed.

**Table 2:** Duck SNP filtration process.

Filtration Steps	Biallelic SNPs	Biallelic Indels	Complex Variants	Total Variants	Filtration Process
1. Genotyping	27,044,733	3,349,438	3,070,748	33,464,919	all genotyping variants
2. Chromosomes	24,676,636	3,113,391	2,575,506	30,365,533	removal variants in unplaced contigs and chromosome W
3. Quality filtration	23,525,876	2,916,668	1,971,707	28,414,251	variant filtration by quality
4. High-quality SNPs	23,049,128			23,049,128	bi-allelic SNPs with call rate ≥0.8 and HWE > 1 × 10^−6^

## Data Availability

Upon reasonable request, the datasets of this study can be made available from the corresponding author. The Illumina Korean Duck SNP panel in this study is available for inquiry.

## Supplementary Materials

The following supporting information can be downloaded at: https://www.mdpi.com/article/10.3390/ani16131995/s1. Table S1: Duck samples and sequencing data used for SNP chip design. Table S2: Candidate duck genomes for SNP chip design. Table S3: QTL-associated genes for Duck bead-chip. Table S4: SNPs in 33 QTL genes (located in genic region). Each row indicates a SNP located in a locus. The IMPACT field indicates the genetic impact calculated by SnpEff. AF means the allelic frequency of the 74 duck samples. Table S5: SNP counts by BIOTYPE. Table S6: SNP counts by IMPACT. Table S7: SNP counts by LOCATION. Table S8: Pairwise Fst. Window size was set to 100 Kb and step size was set to 50 Kb. Figure S1: Average SNP count per gene across chromosomes in the ZJU1.0 genome. The X-axis represents each chromosome, including the Z chromosome, and the Y-axis shows the SNP count per gene. Each blue dot indicates the average SNP density per gene for a given chromosome, with dashed lines highlighting variation. Figure S2: SNP-based genetic distance tree. The tree was computed using the SNPs applied to the duck panel. The right bar indicates the range of breeds: red for Korean Native Duck, green for Peking Duck, blue for Shanma Duck and orange for Shaoxing Duck. The number on each branch indicates the bootstrap value. The bar under the tree is the scale of the branch size.

## Abbreviations

The following abbreviations are used in this manuscript:

NIAS	National Institute of Animal Science
KND	Korean Native Ducks
GBS	Genotyping-by-sequencing
SNP	Single nucleotide polymorphism
NGS	Next-generation sequencing
WGS	Whole-genome sequencing
PKD	Pekin Duck
SMD	Shanma Duck
SXD	Shaoxing Duck
BWA	Burrow-Wheeler Aligner
GATK	Genome Analysis Toolkit
MAF	Minor allele frequency
LD	Linkage disequilibrium
QTL	Quantitative Trait Loci
SRA	Sequence Read Archive
HWE	Hardy–Weinberg equilibrium
