# Supplementary material for: Development and Characterization of a Single Nucleotide Polymorphism Genotyping Panel for Duck Populations
Source: Animals (Basel). 2026 Jun 28;16(13):1995. doi: 10.3390/ani16131995 (PMC13360146; doi:10.3390/ani16131995)
Supplement: Supplementary file 1 [file animals-16-01995-s001.zip › animals-4361768-Supplementary_Figures.pptx]

## Slide 1
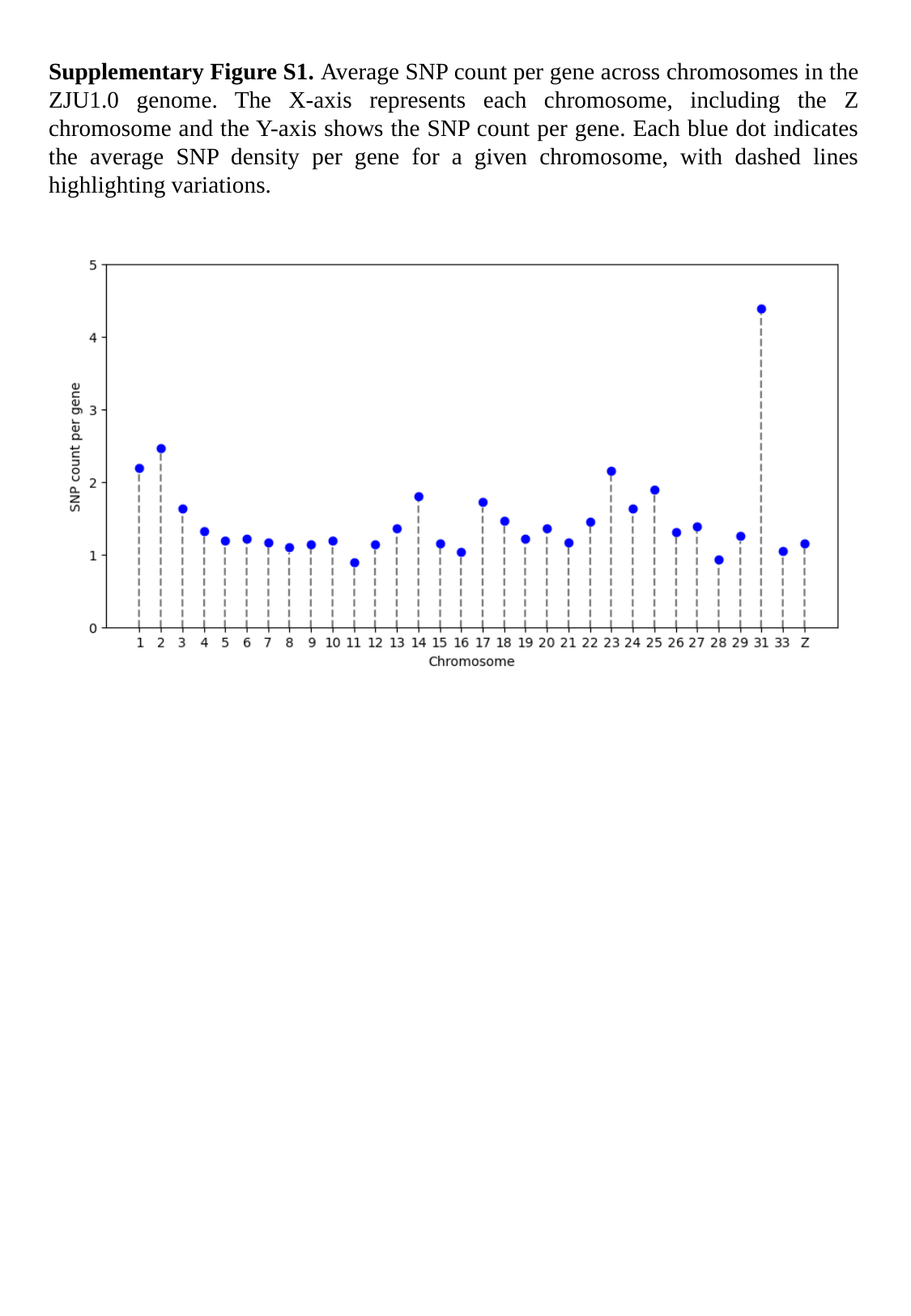

Supplementary Figure S1. Average SNP count per gene across chromosomes in the ZJU1.0 genome. The X-axis represents each chromosome, including the Z chromosome and the Y-axis shows the SNP count per gene. Each blue dot indicates the average SNP density per gene for a given chromosome, with dashed lines highlighting variations.

## Slide 2
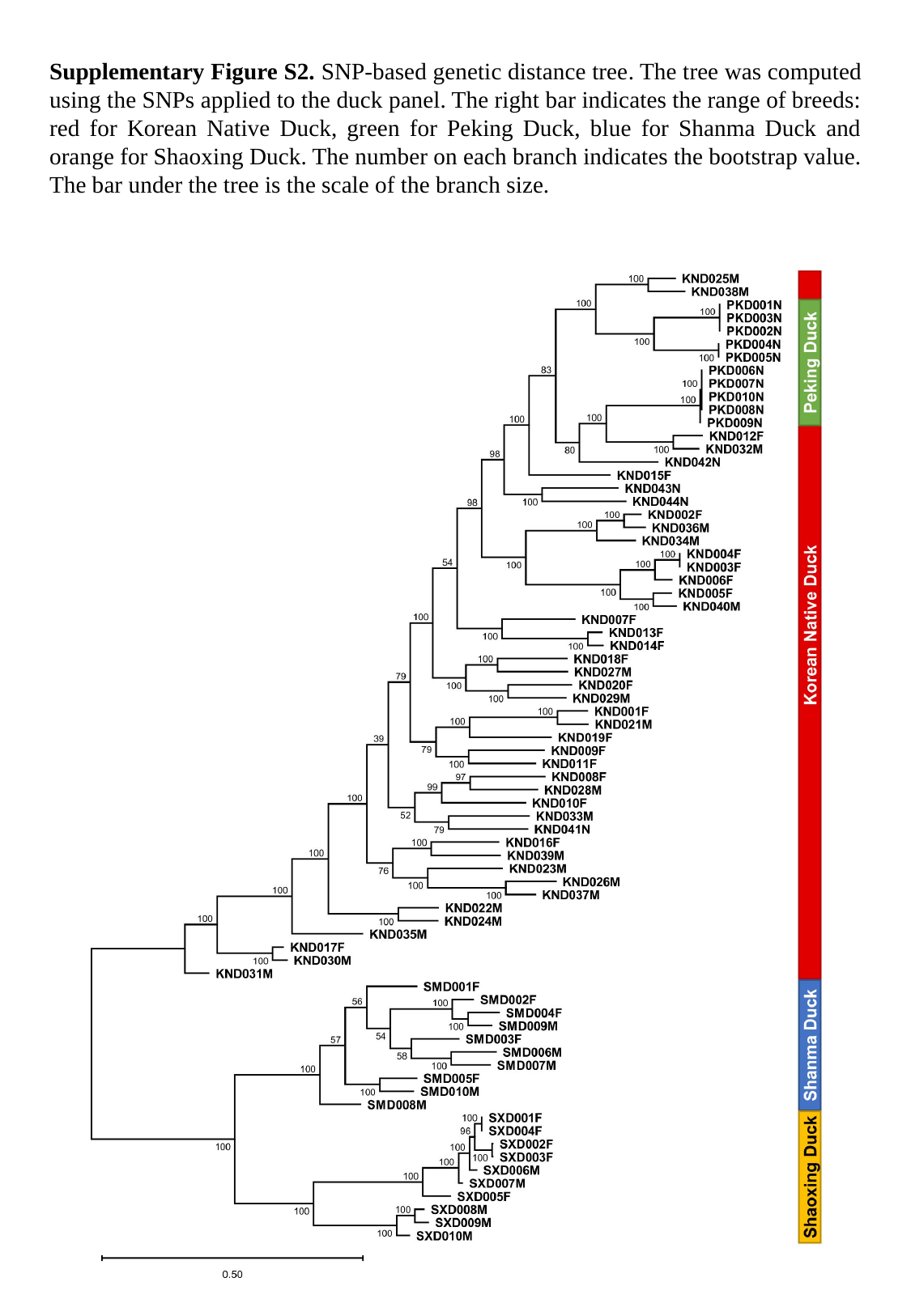

Supplementary Figure S2. SNP-based genetic distance tree. The tree was computed using the SNPs applied to the duck panel. The right bar indicates the range of breeds: red for Korean Native Duck, green for Peking Duck, blue for Shanma Duck and orange for Shaoxing Duck. The number on each branch indicates the bootstrap value. The bar under the tree is the scale of the branch size.
